# Colony size, but not density, affects survival and mating success of alternative male reproductive tactics in a polyphenic mite, *Rhizoglyphus echinopus*

**DOI:** 10.1007/s00265-014-1787-7

**Published:** 2014-10-19

**Authors:** Jacek Radwan, Aleksandra Łukasiewicz, Mateusz Twardawa

**Affiliations:** 1 Evolutionary Biology Group, Faculty of Biology, Adam Mickiewicz University, Poznań, Poland; 2 Institute of Environmental Sciences, Jagiellonian University, Gronostajowa 7, 30-387, Kraków, Poland

**Keywords:** Alternative reproductive tactics, Polyphenism, Conditional strategy, Population density, Population size, Acari, Astigmata

## Abstract

Among acarid mites, a number of species are characterised by the presence of discontinuous morphologies (armed heteromorphs vs. unarmed homeomorphs) associated with alternative mating tactics (fighting vs. scramble competition). In *Rhizoglyphus echinopus*, expression of the fighter morph is suppressed, via pheromones, in large, dense colonies. If this mechanism is adaptive, fighters should have relatively lower fitness in large and/or dense colonies, due to costs incurred from fighting, which is often fatal. In order to test these predictions, we quantified the survival and mating success of fighters and scramblers in colonies of equal sex and morph ratios; these colonies either differed in size (4, 8, or 32 individuals) but not density or differed in density but not size (all consisted of 8 individuals). We found that the relative survival and mating success of fighters was inversely related to colony size, but we did not find a significant effect of colony density. The higher mating success of fighters in small colonies was due to the fact that, after killing rival males, these fighters were able to monopolise females. This situation was not found in larger colonies, in which there was a larger number of competitors and fighters suffered relatively higher mortality. These results indicate that morph determination, guided by social cues, allows for the adaptive adjustment of mating tactics to existing demographic conditions.

**Electronic supplementary material:**

The online version of this article (doi:10.1007/s00265-014-1787-7) contains supplementary material, which is available to authorized users.

## Introduction

Alternative reproductive tactics (ARTs) are among the most spectacular examples of discontinuous phenotypic variation within species (reviewed in: Gross 1996; Brockmann 2001; Shuster and Wade 2003; Oliveira et al. 2008). A common type of ART is found in species that have two distinct male phenotypes: aggressive, often territorial males and sneaker males that avoid direct competition for mates. These alternative behavioural phenotypes often coincide with morphological differences; for example, aggressive males may be armoured with horns (e.g. Emlen 1997a), forceps (e.g. Tomkins and Brown 2004), or sharpened appendages (e.g. Woodring 1969) used in combat, whereas sneaker males are often small in size, which facilitates sneaky behaviours (e.g. Gross 1991; Shuster and Wade 1991; Emlen 1997a). Alternative mating tactics were initially interpreted in terms of the Hawk and Dove evolutionarily stable strategy (ESS) model (Maynard Smith and Price 1973), which led to the prediction that, under certain conditions, genotypes coding for alternative ways of dealing with conflict over resources may co-exist in stable proportions, with equal fitness of alternatives enforced by negative-frequency dependence. However, later models incorporated the observation that individuals typically differ in traits that affect their success in fights (e.g. body size). Given such asymmetry, a conditional strategy in which a male undertakes risky fights only if he is stronger than his opponent (called the “assessor” strategy) is evolutionarily stable (Maynard Smith 1982). Not surprisingly, most cases of alternative mating tactics fit such a conditional strategy model, with less endowed individuals assuming best-of-a-bad-job tactics (Eberhard 1982; Gross 1996). Given the large environmental component to body size variation, expression of alternative tactics may also be influenced by conditions such as food availability during development (e.g. Radwan 1995; Emlen 1997b).

In addition to indirectly causing changes in an individual’s condition, the environment can also influence the success of ARTs by directly affecting the costs and benefits of different tactics. For example, in the acarid mite *Sancassania berlesei*, the success of the aggressive male tactic was shown to be modulated by population size: the aggressive tactic was associated with a high mortality cost, but this cost was counterbalanced by the benefit of better access to females when the number of potential rivals was small (Radwan 1993). Population density has also been implicated as a determining factor in the success of ARTs in species including earwigs (Tomkins and Brown 2004), dung beetles (Moczek 2003; Buzatto et al. 2012), and European bitterlings (Reichard et al. 2004). Similarly, the density of ovipositing females significantly affected the respective success of perching vs. hovering tactics of males in the threadtail damselfly, *Protoneura amatoria* (Larison 2009). Furthermore, the relative success of ARTs among acarids has also been shown to be modulated by the structural complexity of their environment (Lukasik et al. 2006; Tomkins et al. 2011). Here, we investigate whether population size and/or density affects success of ARTs in an acarid mite, *Rhizoglyphus echinopus*.

Acarid mites are a convenient model with which to study ARTs because aggressive fighters can be readily distinguished from benign scramblers by the presence of a thickened, sharply terminated pair of legs that are used to kill rivals by puncturing their cuticle (Woodring 1969; Radwan 1993; Radwan et al. 2000). Although, in all male-dimorphic acarid species that have been investigated thus far, fighter males tend to develop from larger nymphs (Radwan 1995, 2001; Radwan et al. 2002; Smallegange 2011; Tomkins et al. 2011), acarid species differ in how their social environment affects morph determination. In *S. berlesei* and *R. echinopus*, for example, the fighter morph is only expressed in small colonies, while airborne substances (pheromones) emanating from dense colonies can completely suppress its expression (Timms et al. 1980; Radwan 2001; Radwan et al. 2002); in contrast, expression of the fighter morph in *Rhizoglyphus robini* does not depend on colony density during an individual’s nymphal stage (Radwan 1995).

In *S. berlesei*, sensitivity to chemical cues that are correlated with population size has been demonstrated to be adaptive: in large, dense colonies, fighters suffered high combat-related mortality, whereas scramblers were involved in fights less often, survived better, and achieved higher reproductive success (Radwan 1993). The system of pheromonal regulation of morph expression is therefore akin to polyphenisms in which morphology is adaptively modulated by environmental cues, such as protective morphologies induced by cues indicating the presence of predators (reviewed by Roff 1996). In contrast to *S. berlesei*, no significant relationship between population size and relative mortality and reproductive success of male morphs was found in *R. robini* (Radwan and Klimas 2001). Taken together, these results suggest that there is a relationship between pheromone sensitivity and the degree to which the fitness of different ARTs changes in response to population parameters, but data from a larger number of species are necessary to determine the generality of this relationship. Here, we study the effect of population size and density on the survival and mating success of alternative male morphs in *R. echinopus*, which, despite being a congener of *R. robini*, is characterised by pheromonal suppression of the fighter morph (Radwan 2001). Based on this mode of morph determination, we predict that, in *R. echinopus*, males using the fighting tactic will have higher reproductive success than those using the scrambler tactic in small and/or low-density colonies, but the situation will be reversed in favour of scrambler males in large and/or dense colonies.

As a result of very high fecundity and short generation time, populations of acarid mites are usually very large (hundreds to thousands of individuals) and dense, but great variations in population size may occur when the mites colonise new patches of habitat by phoresis. Hypopi are often associated phoretically with dung beetles, and mites were reported to stay on the body of a dead beetle, and to develop and feed on it for some time after its death (Kranz 1978; Houck and Oconnor 1991; Diaz et al. 2000). The number of hypopi found on beetles varies from a few to hundreds (Houck and Oconnor 1991). In this study, we created experimental conditions similar to those occurring when hypopi develop and a number of adult males of approximately the same age compete for females. We first verified the colony size at which fighter tactic expression is suppressed, and then we manipulated colony size and density to understand how this influenced the survival and reproductive success of fighters.

## Methods

### Mites

*R. echinopus* is a broadly distributed acarid mite feeding on bulbs and tubers of various plants and on stored food products (Diaz et al. 2000). Thus, their natural habitat is often patchily distributed, but new patches of habitat are colonised by phoresy, mostly on scarab beetles, but also curculionids and other insects occurring on the same host plants (reviewed by Diaz et al. 2000). The generation time is about two weeks, and includes the following pre-adult stages: egg, larva, protonymph, optional migratory deuteronymph (hypopus), and tritonymph. Females lay hundreds of eggs during lifespan, such that population growth may be high under optimal conditions, leading to depletion of resources, which induces formation of hypopi (Diaz et al. 2000). Here, we follow the classification scheme of genus *Rhizoglyphus* adopted by Manson (1972), that is, the study species has long *Sci* setae and an oval penis base. These features differentiate *R. echinopus* from *R. robini*, which occurs in similar habitats (Diaz et al. 2000).

Mites used in this experiment came from a large stock population originally collected from tulip bulbs and subsequently cultured in the Department of Entomology, SGGW, Warsaw, Poland. The mites were maintained in a controlled temperature chamber at 24 ± 1 °C; to maintain the desired level of humidity in the environment (>90 %), the mites were kept in desiccators filled with KOH solution (153 g/l H_2_O). During the experiments, the mites were reared in tubes, whose bases were made of plaster of Paris mixed with powdered charcoal in order to provide a dark background that facilitates behavioural observations. Tubes were secured with non-absorbent cotton wool and provided with food (powdered yeast) ad libitum. Vials from all treatments were kept in a common large desiccator.

### The choice of colony parameters

**Fig. 1:**
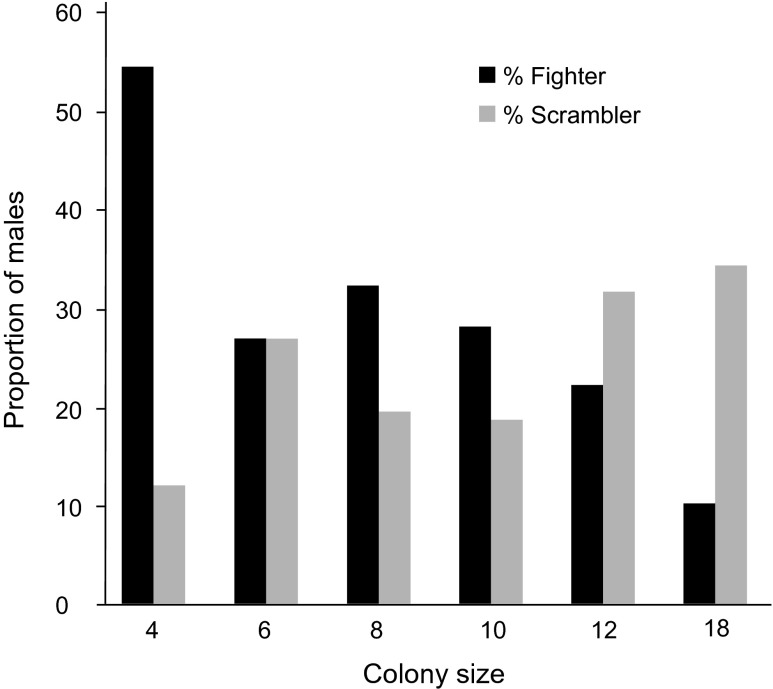
The effect of colony size on the proportions of fighter and scrambler males that emerged in each colony

In order to determine colony parameters which should be used in experiments investigating relative survival and mating success of male morphs, we first determined the proportions of morphs at colonies of different size. To that aim, we placed 4, 6, 8, 10, 12, or 18 protonymphs in tubes (0.8 cm in diameter) and repeated this process 10 times using a total of 60 tubes. Individuals in different tubes were thus exposed to different amounts of pheromones during the sensitive period that determines morph development, which occurs at the early tritonymphal stage (Radwan 2001). Then, during the quiescent stage that precedes the emergence of adults, we isolated tritonymphs into individual cells to prevent fight-related mortality and counted the number of each morph that emerged in each treatment. Consistent with earlier findings (Radwan 2001), the number of nymphs developing into fighters decreased with group size (Fig. 1). For colony sizes between 6 and 12 individuals, both morphs emerged at a similar frequency, so we used a colony size of 8 mites/tube as a benchmark at which we expected the survival and reproductive success of both morphs to be approximately equal. We then created colonies with sizes or densities that were smaller or larger than 8 mites/tube in order to test the prediction outlined in the Introduction that fighters have better survival and higher mating success (relative to scramblers) in small and/or low-density colonies but the situation is reversed in large/dense colonies. We did not replace dead individuals to keep the density/size constant throughout the experiment, as we preferred to create conditions similar to those when new colonies are started by hypopi colonising a new habitat patch, because this is when variation in colony sizes is most likely to occur. Nonetheless, differences in size/density between treatments were large enough to persist throughout experiments.

### The effect of population size and density on survival and mating success of male morphs

Mites for the experiment were obtained from groups of eight protonymphs as described above and were used for the experiments up to 1 day after the final moult. In order to test the effect of population size, we established colonies of three different sizes, but each with a density of about 16 mites/cm^2^, equal sex ratio, and equal proportions of male morphs. Small colonies (*N* = 40) consisted of 4 individuals in 0.55-cm-diameter tubes, intermediate colonies (*N* = 20) of 8 individuals in 0.8-cm-diameter tubes, and large colonies (*N* = 10) of 32 individuals in 1.6-cm-diameter tubes.

In order to test the effect of population density, colonies of eight individuals (four females, two fighters, two non-fighters) were established in cells that measured 0.55 cm (*N* = 18), 0.8 cm (*N* = 20) and 1.6 cm (*N* = 20) in diameter, which resulted in three density treatments: 34, 16, and 4 mites/cm^2^, respectively.

In both the population size manipulation experiment and density manipulation experiment, we recorded daily survival and the number of copulations and fights by males three times a day between 9 am and 6 pm at 4 h intervals. Such an interval prevented scoring the same behaviour more than once, as both mating and fights do not last longer than a couple of hours (personal observations). The observations were carried out until new generation emerged, i.e. for 13 days in size manipulation experiment and 15 days in density manipulation experiment.

### Data analysis

Our aim was to compare fitness components of fighters relative to that of scramblers between colonies of different size or density. Therefore for each colony, we calculated two measures of relative success of male morphs: relative survival and relative mating success. The relative survival of fighters compared to scramblers was calculated as the number of days survived summed for all fighters in a given colony, divided by the summed number of days survived for scramblers. Given equal numbers of males of both morphs at the beginning of the experiment, higher relative survival of fighters implies that during the time of the experiment, i.e. before the new generation matured, an average fighter survived better and thus had more opportunities to inseminate females. Similarly, the relative mating success of fighters was calculated as (number of copulations by fighters in a colony + 1)/(number of copulations by scramblers in a colony + 1); the value of 1 was added to the number of mating attempts because, in some cases in small colonies, no copulations by scramblers were recorded. The relative survival and mating success was compared between population size treatments and between density treatments using a non-parametric Kruskal-Wallis test. To check if fights between fighters and between fighters and scramblers occurred with equal probability, we compared observed proportions to the expectation given by the number of fighters (f) and scramblers (s) in a colony, i.e. $$ \left(\begin{array}{c}\hfill f\hfill \\ {}\hfill 2\hfill \end{array}\right)/\left[\left(\begin{array}{c}\hfill f\hfill \\ {}\hfill 2\hfill \end{array}\right)+ fs\right] $$ (Radwan 1993).

## Results

Male mortality was much higher than female mortality in both experiments (Table 1; Table S1), a result that was most likely due to fighting between males. Indeed, when there was only one fighter in a colony, fighter mortality was very low and similar to that of females (Table 1; Table S1, small colonies). Fighter survival decreased with colony size (Table 1, *H* = 48.5, *N* = 79, *P* < 001), but that of scramblers did not (*H* = 1.1, *N* = 70, *P* = 0.571). Interestingly, survival of fighters, but not scramblers increased with colony density (Table 1; fighters, *H* = 14.9, *N* = 58, *P* = 0.001; scramblers, H = 2.2, B = 58, *P* = 0.324).

**Table 1:** Number of days survived by males (based on colony averages) in colonies differing in size (small, 4 mites; medium, 8 mites; large, 32 mites) or density (8 mites at densities of 34, 16, and 4 mites/cm^2^)

Colony type (*N*)		Mean (SE)			Median (range)		
		Females	Fighters	Scramblers	Females	Fighters	Scramblers
Size	Small (40)	12.7 (0.14)	12.8 (0.25)	9.5 (0.64)	13 (8–13)	13 (8–13)	13 (1–13)
	Medium (20)	12.8 (0.20)	8.9 (0.35)	9.7 (0.91)	13 (11–13)	7.3 (7–13)	8.5 (6–13)
	Large (10)	19.9 (0.28)	8.1 (0.50)	9.9 (1.29)	13 (12.3–13)	7.8 (5.8–11.2)	10 (5.8–13)
Density	Low (20)	14.7 (0.21)	8.72 (9.69)	11.2 (0.70)	15 (11.5–15)	8 (4–15)	10 (6.6–15)
	Medium (20)	14.5 (0.21)	10.9 (0.69)	11.4 (0.70)	15 (12–15)	9.3 (8–15)	11.3 (8–15)
	High (18)	14.2 (0.22)	12.5 (0.72)	12.7 (0.74)	15 (11.5–15)	8 (4–15)	10 (6.5–15)

For size manipulation, maximum number of days was 13, for density manipulation 15

In 17 small colonies, a fighter killed its rival and monopolised females (Table S1). In intermediate-sized colonies, monopolisation occurred in only one colony, and no cases of monopolisation were recorded in large colonies. In the population density treatment (equivalent in size to intermediate colonies), monopolisation by a fighter was recorded in four low-density colonies. In one high-density colony, both fighters died by the end of the experiment.

Fights were observed both between pairs of fighters and between fighters and scramblers (Table 2). The total number of fights observed was low and most of them occurred during the first day of observations, so we only analysed data from that day. We compared frequencies of fights between pairs of fighters and between fighters and scramblers for large colonies, and for medium-size colonies pooled for all densities (as the frequencies did not differ significantly between densities, Yates *χ* ^2^ = 0.079, df = 2, *P* = 0.961). In both cases, fights between fighters occurred disproportionately more often (large colonies, *χ* ^2^ = 7.125, df = 1, *P* = 0.007; medium-sized colonies, *χ* ^2^ = 15.244, df = 1, *P* < 0.001) than expected (Table 2). Likewise, when we pooled data from all colonies that contained eight individuals at medium density across both treatments, we also found that conflicts between fighters occurred significantly more often than expected (*χ* ^2^ = 6.612, *P* = 0.010).

**Table 2:** Number of fights that occurred between pairs of fighters (f-f) and pairs of fighters and scramblers (f-s) observed in colonies differing in size and density; the proportion of fights expected between fighters given the number of fighters (f) and scramblers (s) in a colony was $$ \left(\begin{array}{c}\hfill f\hfill \\ {}\hfill 2\hfill \end{array}\right)/\left[\left(\begin{array}{c}\hfill f\hfill \\ {}\hfill 2\hfill \end{array}\right)+ fs\right] $$, i.e., 0.45 in large colonies (32 individuals) and 0.375 in medium-sized colonies (8 individuals); for small colonies, containing one fighter only, f-f fights do not apply (n.a.)

Colony size				Colony density			
		f-f	f-s			f-f	f-s
Small	Day 1	n.a.	10	Low	Day 1	6	4
	Total	n.a.	11		Total	7	9
Intermediate	Day 1	10	5	Medium	Day 1	8	4
	Total	11	10		Total	16	17
Large	Day 1	10	2	High	Day 1	4	1
	Total	19	7		Total	12	9

**Fig. 2:**
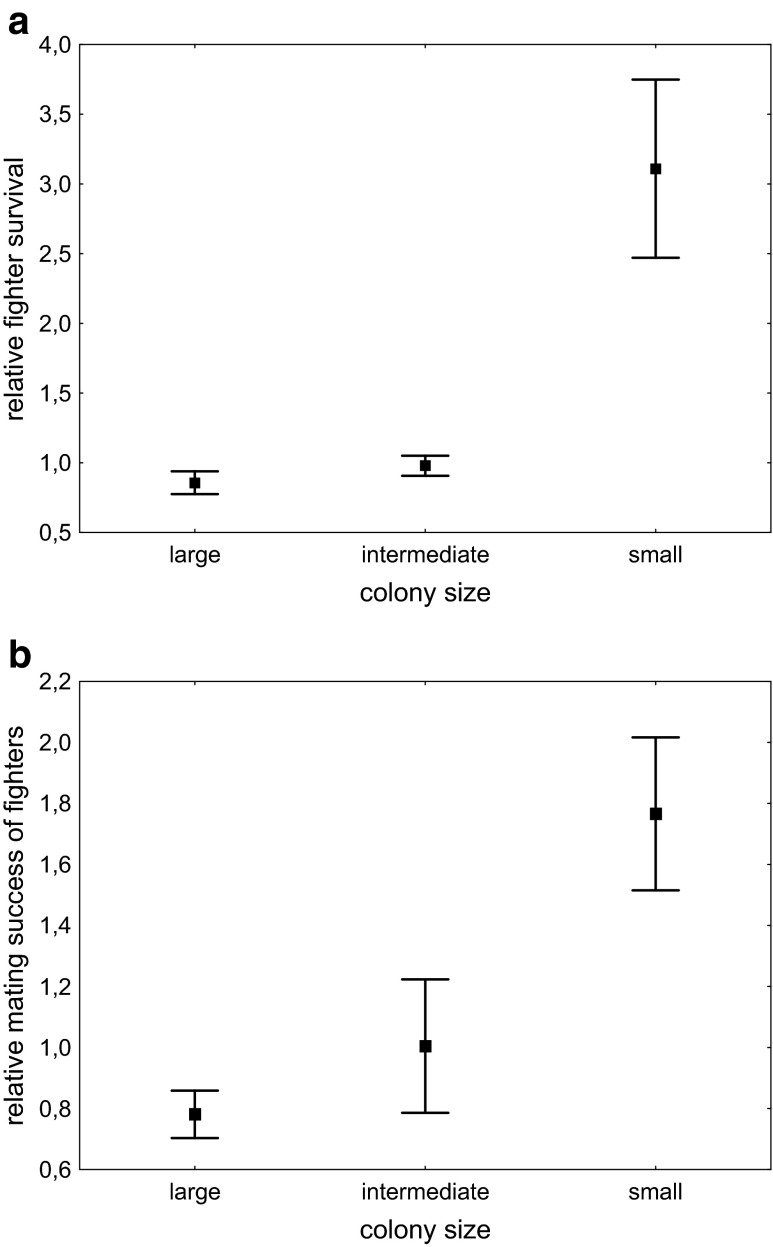
The effect of colony size on fighters’ **a** survival and **b** mating success relative to scramblers. *Error bars* denote ± SE

Colony size had a significant effect both on the relative survival of fighters (Kruskal-Wallis *H* = 15.5, *N* = 70, *P* < 0.001) and on their relative mating success (Kruskal-Wallis *H* = 10.2, *N* = 70, *P* = 0.006). Relative fighter survival and mating success were highest in the smallest colonies (Fig. 2).

**Fig. 3:**
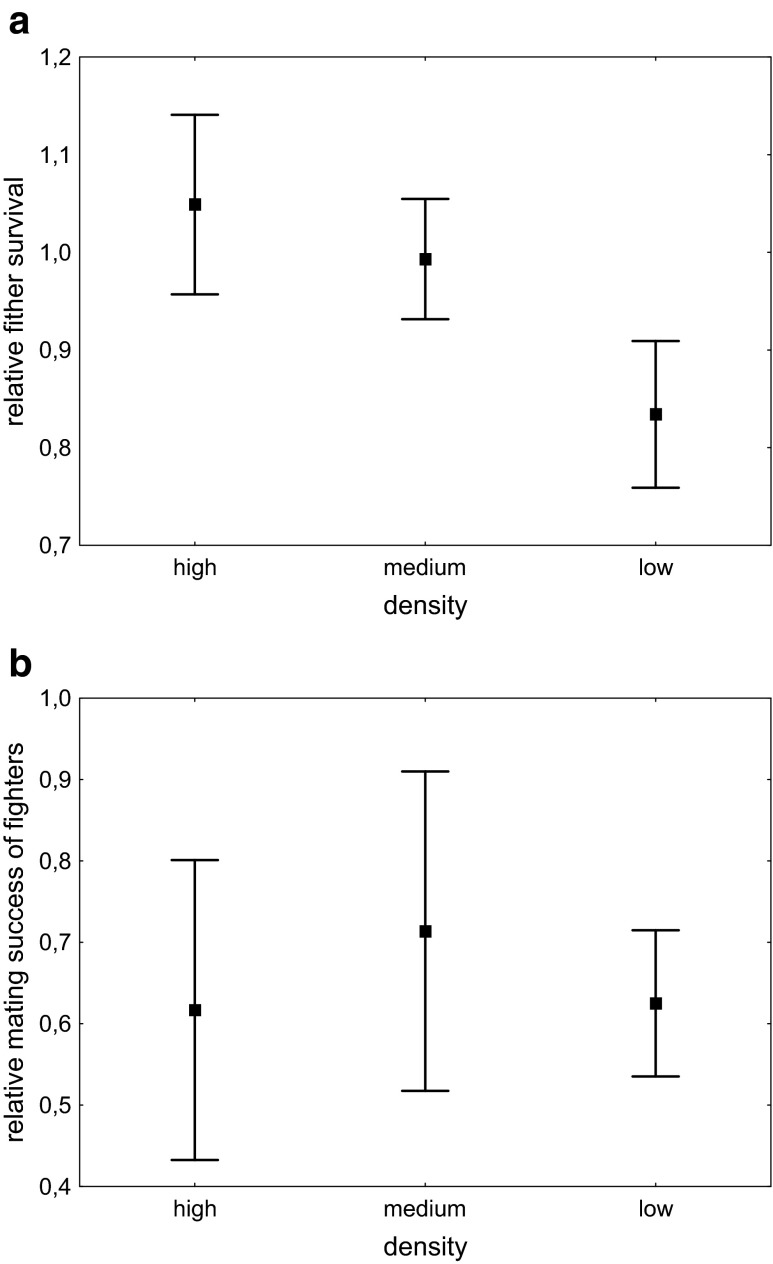
The effect of colony density on fighters’ **a** survival and **b** mating success relative to scramblers. *Error bars* denote ± SE

The relative survival of fighters tended to increase with colony density (Fig. 3a), but this effect was not statistically significant (Kruskal-Wallis *H* = 5.0, *N* = 58, *P* = 0.082). Density did not significantly affect the relative mating success of fighters (Kruskal-Wallis *H* = 1.5, *N* = 58, *P* = 0.485) (Fig. 3b).

## Discussion

Population parameters, such as size and density, are thought to play an important role in shaping the relative success of various ARTs (Radwan 1993; Knell 2009). For example, studies using correlational data on population density and horn allometry across several populations of the dung beetle (*O. taurus*) showed that the frequency of horned major males decreased at high population densities as a result of a change in the allometric scaling of horn size with body size (Moczek et al. 2002; Moczek 2003). This result is consistent with the hypothesis that sneaker tactics are adaptive and enhance reproductive success in higher-density populations. In contrast, in their study of earwigs, Tomkins and Brown (2004) proposed a different explanation: in high-density populations, males with superior fighting ability will be favoured because of the increased likelihood of encounters with other males. In both dung beetles and earwigs, though, the effect of density on the relative success of different ARTs has not yet been demonstrated. In an experimental study of European bitterlings, Reichard et al. (2004) manipulated the density of males and demonstrated that, at low densities, territorial males have a considerable fitness advantage over sneakers; however, at high densities, both alternative mating tactics had similar levels of success. Here, we have demonstrated that, in *R. echinopus*, colony size, rather than density, affects the relative success of ARTs.

Given that in *R. echinopus* fighter morph expression is suppressed as population size increases (Radwan 2001; Fig. 1), our findings are consistent with the idea of adaptive developmental plasticity in ART expression: when the relative success of different ARTs depends on an environmental factor, and it is possible to detect cues that predict the environment in which certain ARTs will be expressed, developmental processes should evolve to achieve adaptive plasticity in ART expression. The fighter tactic was effective only when the population size was small because only under such conditions was it possible to monopolise females by killing rival males. In larger colonies, fighter tactics were associated with higher mortality and lower mating success. The higher mortality was most likely a result of the propensity of fighters to engage in combat with other males, which often resulted in death. Similar findings have been previously reported for another acarid, *S. berlesei* (Radwan 1993). Likewise, in dung beetles, anticipated increases in population density, which is believed to shape the relative success of horned and hornless males, has been recently demonstrated to affect the male phenotype via parental effects (Buzatto et al. 2012).

The period during which acarid mites are sensitive to cues regarding population size is also consistent with the hypothesis of adaptive plasticity. This period is the early tritonymphal stage, which follows the migratory, facultative deuteronymph stage. Radwan (1993) argued that conditions favouring fighter males occur mostly when a small group of deuteronymphs, which are phoretic on insects, start a new colony. Due to their high reproductive output, acarid colonies grow rapidly, and conditions favouring fighters are probably rarely present past the early migration stage. The high dynamics of populations of acarids also suggest that resources are depleted quickly, and as a result, migration events could occur often enough to exert selective pressure favouring the expression of the fighter phenotype.

Radwan (1993) argued that population size, not density, determines the relative success of fighter males in *S. berlesei*, as fighters succeed only if the colony size is small enough that they can kill all their rivals and subsequently monopolise females. However, the experimental design of his study confounded size and density. Here, we have separated the effects of colony size and density and have confirmed that colony size, but not density, affects the relative fitness of male morphs in *R. echinopus*. We had initially predicted that fighters would survive better than scramblers under low-density conditions but worse in high-density conditions, but, in fact, the relative survival of fighters tended to increase, rather than decrease, with colony density (Fig. 3b). Likewise, fighter mortality was the lowest at the highest density. Further work utilising more intensive scoring of behaviours should reveal whether this effect could be due to decreased fighter aggression. Such behavioural plasticity may be adaptive, preventing fighters from performing costly behaviours under unfavourable conditions: high density would normally be correlated with large population size where fighter tactic is disfavoured, as shown in this study.

Under demographic conditions in which their fitnesses are approximately equal, both fighter and scrambler morphs are expected to occur. Indeed, we observed approximately the same degree of survival and mating success of each morph in groups of eight mites maintained in 0.8-cm-diameter cells (a condition that resulted in both morphs occurring in similar proportions; Fig. 1). When tactics co-occur, their relative fitness may be related to body size; for example, adopting aggressive tactics could be more beneficial to larger and stronger males (Maynard Smith 1982; Gross 1996). Consistent with this idea is the observation that fighter males in *R. echinopus* emerge from heavier nymphs (Tomkins et al. 2011); the same is true in *S. berlesei* (Radwan et al. 2002). It thus appears that adaptive morph determination in these species may involve a complex interplay between responses to external demographic cues and internal cues of body condition. In this study, we demonstrated that the former mechanism does contribute to the maintenance of alternative male morphs in *R. echinopus* by causing the reversal of fitness ranking of the alternatives with increasing population size. However, the adaptive significance of the latter mechanism remains to be investigated.

We used equal proportion of males in our experiments, therefore we cannot exclude that relative success of male morphs is additionally affected by frequency dependence; for example, in large populations fighters would probably not suffer costs of fights if they occurred in low frequencies, such that encounters with other fighters would be rare. However, the benefits of monopolisation would not occur either, and in fact in large stock populations, fighter males do not occur (personal observations). Furthermore, in a congener species, *R. robini*, mating success of male morphs did not depend on their proportions in a colony (Radwan and Klimas 2001).

Previous work on two other species of acarids has shown that in *S. berlesei*, a species in which male morph expression is guided by pheromonal cues linked to population size, the relative success of fighters depends on population size (Radwan 1993), whereas in *R. robini*, neither morph expression nor fitness is related to population size. This suggests that the reaction norms that guide morph expression may evolve in response to a change in selective pressures on morph expression under varying demographic conditions and thus vary among species in an adaptive fashion. Our results lend support to this hypothesis by showing a pattern similar to that found in *S. berlesei*, a congener of *R. robini*: sensitivity to pheromones is associated with the reversal of the relative success of fighters and scramblers that occurs as population size increases.

At population densities at which both morphs are expressed, the degree of fighter morph expression has been shown to respond quickly to experimental selection in both *S. berlesei* (Unrug et al. 2004) and *R. echinopus* (Tomkins et al. 2011). Such changes could be due to a change in mite sensitivity to pheromones and/or the change in the reaction norm that translates body size into the probability of fighter expression. Irrespective of the mechanism, the proportion of fighters expressed at a given population density was observed to evolve rapidly. Similarly, populations of *S. berlesei* collected from different locations and held in common-garden conditions have been shown to differ in the proportion of fighters emerging at various colony sizes (Tomkins et al. 2004), implying genetic variation for this reaction norm. Furthermore, a study of experimental evolution in *R. echinopus* showed that when a change in environmental complexity caused a shift in the relative costs and benefits of expressing the fighter phenotype, the reaction norm evolved to decrease the probability of expressing the fighter phenotype in an unfavourable habitat (Tomkins et al. 2011). A genetic basis for variation in the threshold of responses to cues inducing phenotypically plastic alternative phenotypes has previously been documented in contexts other than that of sexual selection, such as in cases of predator-induced defences (Hazel and West 1982) or migratory polymorphism (Fairbairn and Yadlowski 1997; Knulle 2003). The existence of standing genetic variation for sensitivity to pheromone cues should facilitate the evolutionary adjustment of morph expression patterns to changes in benefits of ARTs under different colony sizes.

Given that *R. echinopus* is considered to be closely related to *R. robini*, and the two species live in similar habitats (Manson 1972; Diaz et al. 2000), it remains unclear why the success of alternative male morphs in *R. robini* does not depend on population size (Radwan and Klimas 2001). Despite morphological similarity to *R. echinipus*, *R. robini* is generally smaller and has slightly shorter and stouter legs (personal observations). Radwan and Klimas (2001) proposed that the lower mobility associated with such features may make scrambler males of *R. robini* less capable of escaping attacks from fighters, thus removing the survival advantage in large colonies that would be experienced by the more agile *S. berlesei* or *R. echinopus*. However, this speculation has not yet been empirically tested.

## Electronic supplementary material

Below is the link to the electronic supplementary material.

ESM 1 (DOC 75 kb)
