# Supplementary material for: Colony size, but not density, affects survival and mating success of alternative male reproductive tactics in a polyphenic mite, Rhizoglyphus echinopus
Source: Behav Ecol Sociobiol. 2014 Oct 19;68(12):1921–8. doi: 10.1007/s00265-014-1787-7 (PMC4220114; doi:10.1007/s00265-014-1787-7)
Supplement: Supplementary file 1 — (DOC 75 kb) [file 265_2014_1787_MOESM1_ESM.doc]

| Colony type (n) | | Mean (SE) | | | Median (range) | | |
| --- | --- | --- | --- | --- | --- | --- | --- |
|  | | females | Fighters | scramblers | females | fighters | scramblers |
| Size | Small (40) | 0.04 (0.02) | 0.03 (0.02) | 0.38 (0.08) | 0 (0-0.5) | 0 (0-1) | 0 (0-1) |
|  | Medium (20) | 0.05 (0.02) | 0.4 (0.05) | 0.35 (0.06) | 0 (0-0.25) | 0.5 (0-0.5) | 0.5 (0-1) |
|  | Large (10) | 0.03 (0.01) | 0.63 (0.04) | 0.38 (0.06) | 0 (0-0.06) | 0.6 (0.3-0.8) | 0.4 (0-0.6) |
| Density | Low (20) | 0.06 (0.03) | 0.53 (0.06) | 0.38 (0.06) | 0 (0-0.5) | 0.5 (0-1) | 0.5 (0-1) |
|  | Medium (20) | 0.09 (0.03) | 0.38 (0.05) | 0.33 (0.05) | 0 (0-0.25) | 0.5 (0-0.5) | 0.5 (0-0.5) |
|  | High (18) | 0.11 (0.04) | 0.22 (0.07) | 0.19 (0.06) | 0 (0-0.5) | 0 (0-1) | 0 (0-0.5) |
